# An innovative `8'-shaped deep-layered suture method for incision suture rejection: a case report

**DOI:** 10.1093/jscr/rjag041

**Published:** 2026-02-05

**Authors:** Bin-Yu Wang, Zhi Wang, Mi Yuan, Yun-Cang Wang

**Affiliations:** Department of Thoracic Surgery, Hospital of Chengdu Office of People's Government of Xizang Autonomous Region (Hospital. C.X.), No. 20 Ximianqiao Heng Street, Jiangxijie Sub-district, Wuhou District, Chengdu 610041, Sichuan Province, P.R. China; Department of Thoracic Surgery, Hospital of Chengdu Office of People's Government of Xizang Autonomous Region (Hospital. C.X.), No. 20 Ximianqiao Heng Street, Jiangxijie Sub-district, Wuhou District, Chengdu 610041, Sichuan Province, P.R. China; Department of Thoracic Surgery, Hospital of Chengdu Office of People's Government of Xizang Autonomous Region (Hospital. C.X.), No. 20 Ximianqiao Heng Street, Jiangxijie Sub-district, Wuhou District, Chengdu 610041, Sichuan Province, P.R. China; Department of Thoracic Surgery, Hospital of Chengdu Office of People's Government of Xizang Autonomous Region (Hospital. C.X.), No. 20 Ximianqiao Heng Street, Jiangxijie Sub-district, Wuhou District, Chengdu 610041, Sichuan Province, P.R. China

**Keywords:** '8′-shaped deep-layered suture method, innovation, incision suture rejection, surgical technique

## Abstract

To report a patient with incision suture rejection who was successfully treated using the innovative `8'-Shaped Deep-Layered suture method. A 33-year-old female patient presented with a severe suture rejection，the incision had dehisced and was unable to heal. The innovative suturing method was applied, involving the use of 2–0 absorbable sutures in an `8'-Shaped Deep-Layered suture method in the deep layers (muscle and subcutaneous tissue layers). Once the wound had healed, the sutures in the subcutaneous and muscle layers were completely removed in a single procedure from the outside of the skin. The `8'-Shaped Deep-Layered suture method is an effective innovative technique that overcomes the limitations of traditional suturing methods and effectively prevents incision suture rejection in patients.

## Introduction

Surgery currently stands as an important, and in many cases, the sole method for treating numerous diseases, including trauma, tumors, infections, and deformities. Suturing is one of the most fundamental techniques in surgical procedures, serving purposes such as hemostasis, traction, and tissue reconstruction [1]. Sutures facilitate tissue healing by offering tension support and approximating tissues for a specified duration. The healing process of an injury can be divided into three stages: the inflammatory stage, the proliferative stage, and the maturation stage. However, in reality, each stage overlaps and progresses sequentially with the others [2]. For abdominal wall incisions, whether layered or full-thickness sutures are used, the healing parenchyma involves tissue scar healing [3].

Suture rejection is a type IV (cell-mediated immune) allergic reaction, which is closely related to the patient's constitution and is difficult to predict in clinical practice [4]. In China, surgical sutures have traditionally been predominantly made from silk thread. Clinically, common rejection reactions to sutures often involve the rejection of silk thread. Loop rejection reactions may occur frequently and may or may not be associated with infection. The tissues surrounding the thread may experience repeated effusion, empyema, and skin redness and swelling. The occurrence time varies from a few hours to several years after the operation. Often, after the rejection reaction at one suture site is cured by removing the suture, another rejection reaction occurs at a different site after an interval of time [5]. The clinical manifestations are not exactly the same, some have only local mild discomfort, some show strong rejection, local obvious redness, swelling, heat, pain and other inflammatory reactions, some even prolonged until many years later the suture thread is completely discharged or removed, and some cause local scar hyperplasia affecting the esthetics [6].

Surgical removal of all rejected threads in the subcutaneous tissue is the main treatment method. Most patients' wounds can heal quickly after the threads are removed. Some patients require secondary suturing due to wound dehiscence. Absorbable sutures are currently used for secondary sutures. Although absorbable sutures can theoretically be absorbed by themselves, absorbable sutures made of some materials sometimes have a greater rejection reaction than silk threads, and the treatment is still not ideal.

## Case report

**Figure 1:**
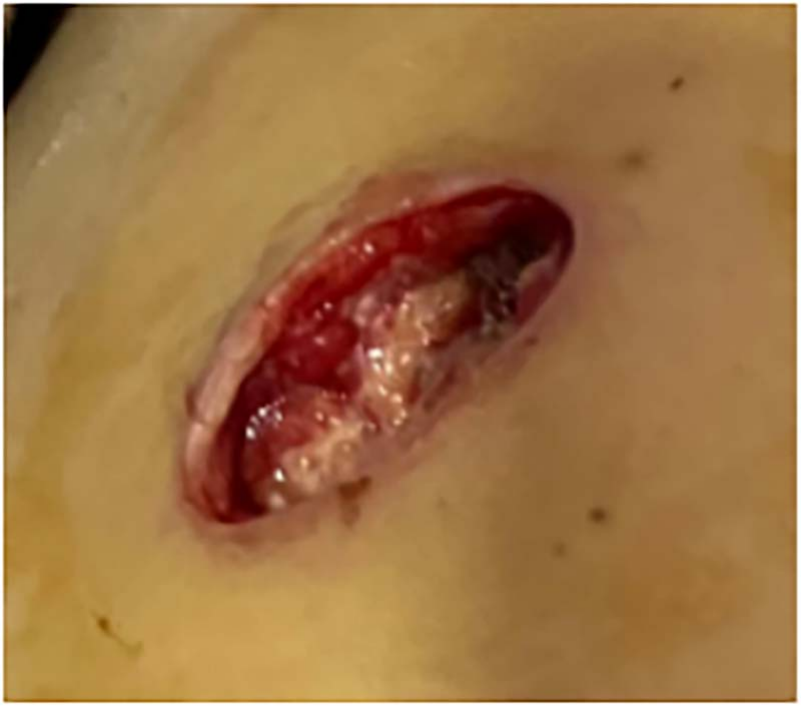
Wound dehiscence.

**Figure 2:**
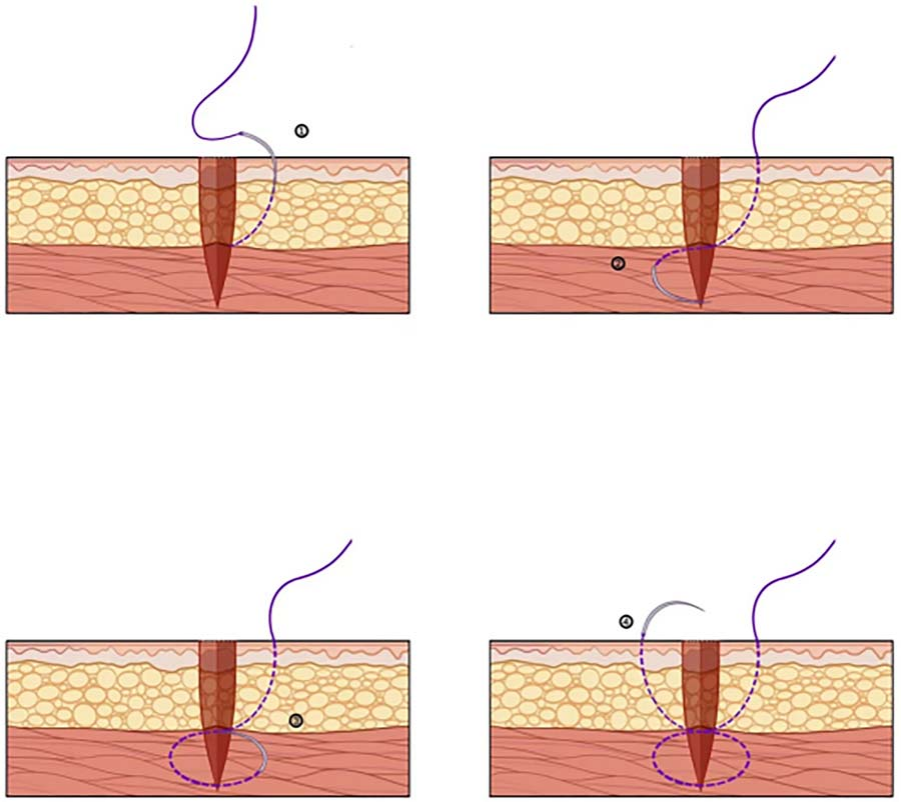
Needle insertion sequence during suturing.

**Figure 3:**
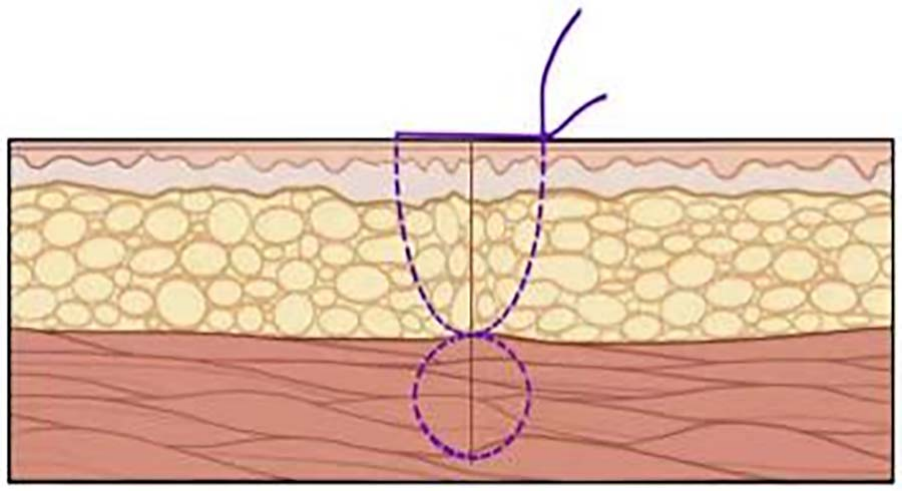
Incision condition after suturing.

**Figure 4:**
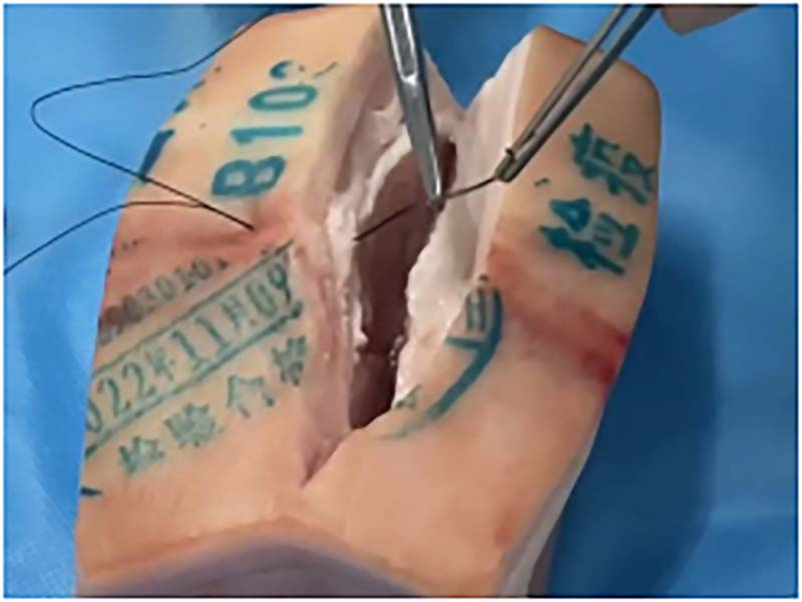
Stitching experiment on pork.

**Figure 5:**
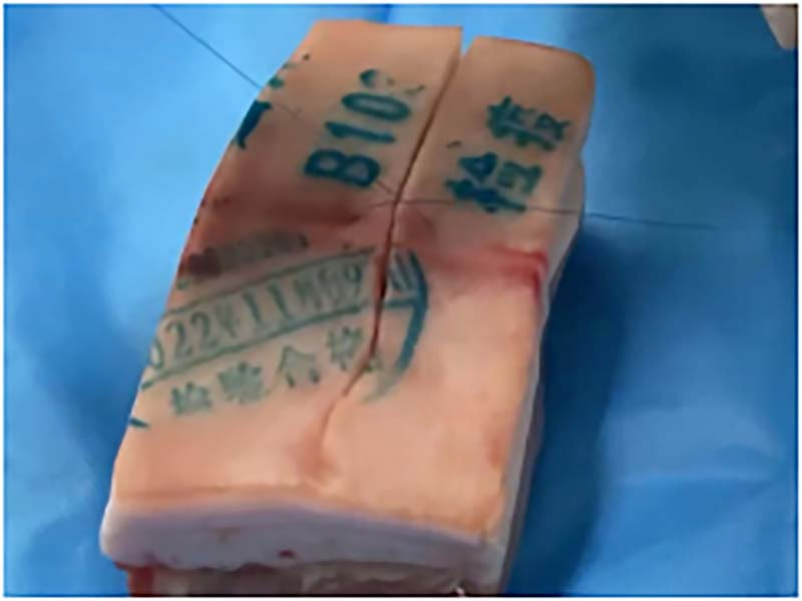
This stitching method on pork is good.

**Figure 6:**
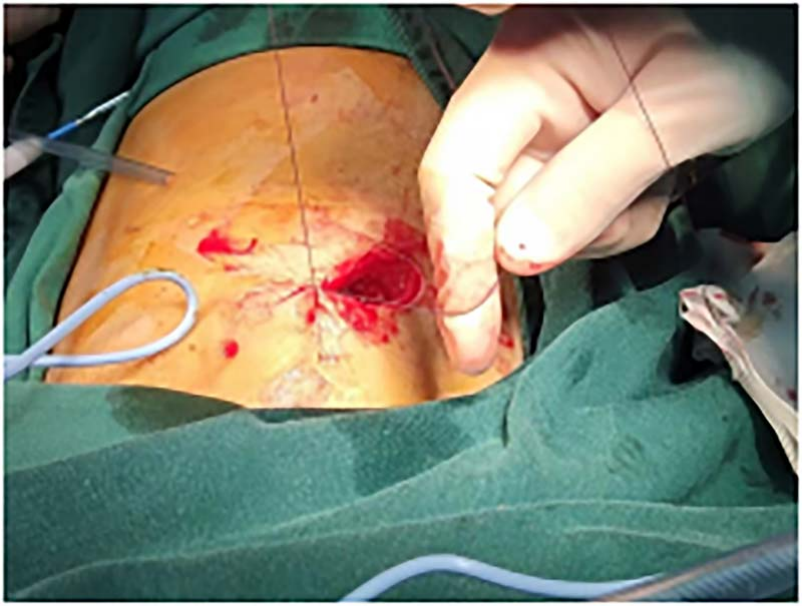
Use absorbable sutures to perform `8' deep-layered suture.

**Figure 7:**
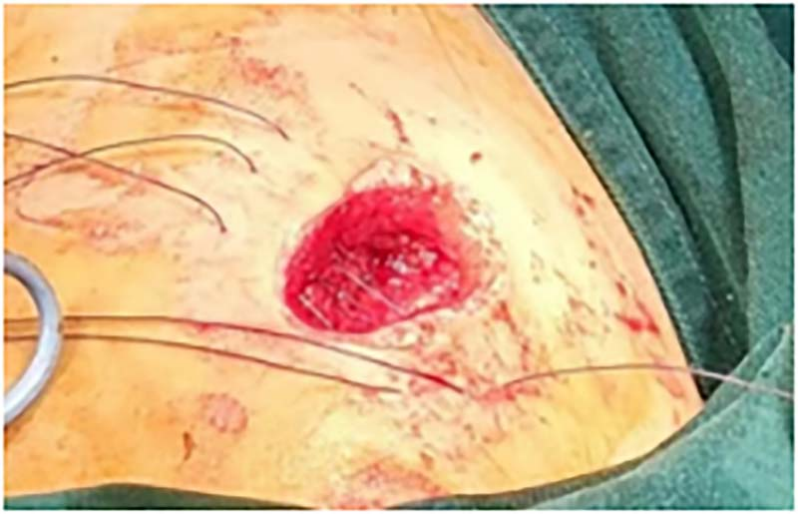
Neatly arranged sutures.

**Figure 8:**
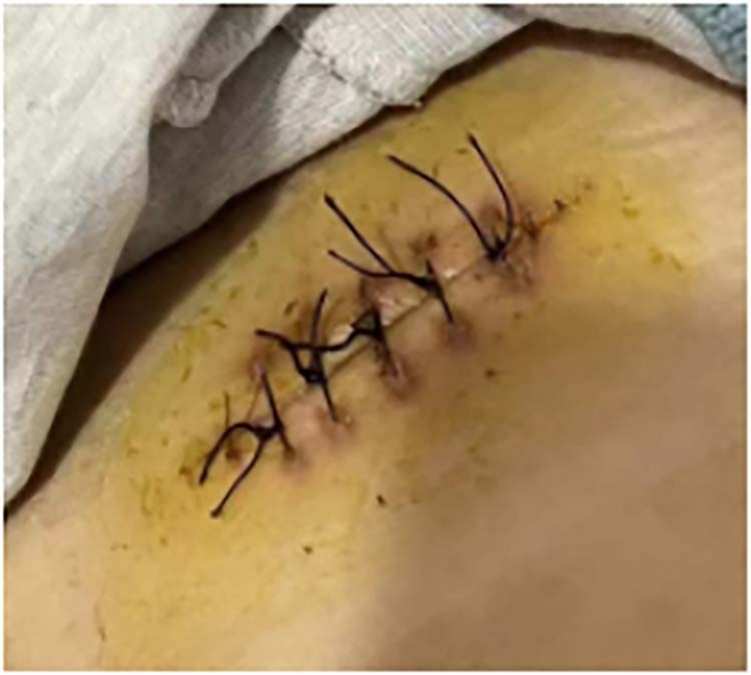
Situation after wound suturing.

**Figure 9:**
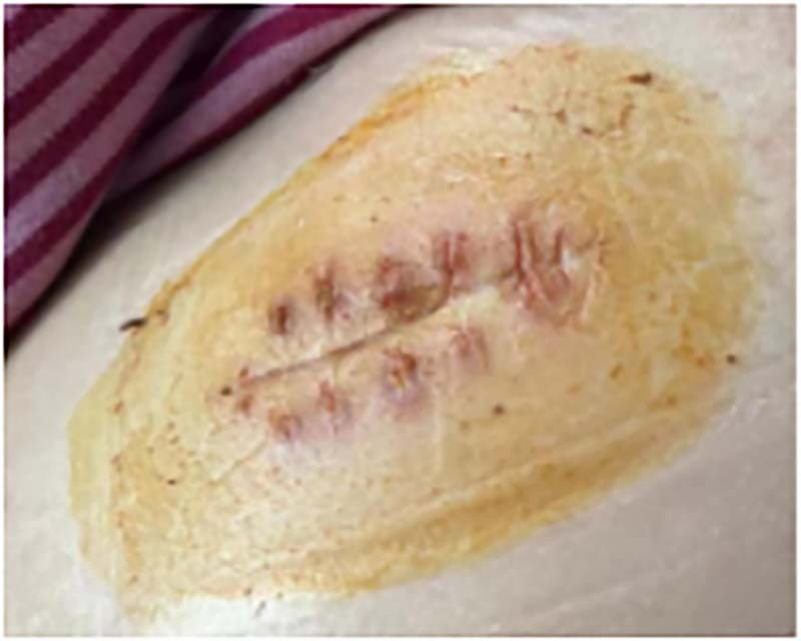
Condition after wound healing.

This is a 33-year-old female patient was admitted due to ‘Postoperative wound dehiscence and infection 10 days after surgery for left lower lobe adenocarcinoma’. The patient developed postoperative incision redness, swelling and pain, which were not significantly improved after dressing change in the local hospital. During this period, the patient was treated with anti-infection and local drainage and dressing change, without significant improvement. The patient visited our hospital, and was admitted to our department as ‘postoperative wound infection’ in the outpatient department. Physical examination showed a surgical incision about 6 cm in length and 3 cm in depth on the left chest wall, with local redness, swelling and dehiscence (Fig. 1). After active dressing change, suture removal and drainage, wound rejection was reduced, but due to too large and deep incision, it could not heal spontaneously. A second suture was planned and given the rejection constitution of the patient, replacement of absorbable suture also had the possibility of rejection. Therefore, it is feasible to innovatively complete the method of `8'-Shaped Deep-Layered suture (Figs 2 and 3), and perform suture experiment and suture removal experiment on isolated pork (Figs 4 and 5). Using this innovative suture method, we performed a secondary suture on the patient with 2–0 absorbable suture (Figs 6–8). After about a month, the wound fully healed and we removed the subcutaneous and muscle layer sutures from the outside of the skin in one go (Fig. 9).

## Discussion

1. Common influencing factors of suture rejection.

Common influencing factors of suture rejection in clinical practice The influencing factors of suture rejection are as follows [7]: (i) Poor incision apposition, different skin height on both sides, some knots higher than the lower side of the subcutaneous or dermal, and even close to the epidermis or knots exposed, resulting in knots located in the skin, accounting for more than half of the clinical cases, especially the final suture is very rough in those with longer operation time; (ii) large incision tension, large scar, thick suture, especially those who tie three knots, thick suture and large knots are more common in the knots removed from clinical cases; (iii) medical silk is protein fibers obtained from silkworm saliva, knitted after staining treatment, with good strength, firm and easy operation, but poor degradation retention time, multiple threads gap can well hide bacteria, can cause tissue bacterial inflammation or non-bacterial inflammatory reactions and rejection.

2. Common treatment methods for suture rejection have been reported to cause.

It has been reported that deep tissue reactions have led to intestinal perforation [8]. Therefore, for patients with recurrent knot reactions, the knots and hyperplastic granulation tissue should be removed in a timely manner, and absorbable stitches should be used for suture during reoperation. For rapidly growing tissues or deep tissues, absorbable stitches should be used for suturing as much as possible to reduce the serious consequences caused by the inability to handle deep thread knots in a timely and effective manner after rejection occurs [9, 10].

3. Characteristics of this innovative suture method and thinking.

About the application of `8'-Shaped Deep-Layered suture in this patient This innovative method, in other studies mainly focus on the improvement of suture, carry out the exploration study on the suture method, breaking through the limitations of the traditional suture method. This innovative suture method is to suture the subcutaneous tissue of the skin and the muscular layer in two layers by inserting the needle lead in depth of the word `8' to maintain a clear hierarchical healing. When the wound is healed, remove the suture from the skin to pull out the suture of two tissue layers completely, and there is no suture residue in subcutaneous tissue and muscle layer, avoiding the recurrence of suture rejection.

To verify the feasibility, the investigators performed suture experiments and suture removal experiments on ex vivo pork. This method was successful in secondary closure of the patient’s wound.

## Conclusion

The `8'-Shaped Deep-Layered suture is a good innovative suturing method that breaks through the limitations of traditional suturing methods and effectively avoids suture rejection in patients again.
